# Impact of Lifestyle Modifications on Cardiovascular Health: A Narrative Review

**DOI:** 10.7759/cureus.42616

**Published:** 2023-07-28

**Authors:** Gunjan K Ghodeshwar, Amol Dube, Deepa Khobragade

**Affiliations:** 1 Cardiology, All India Institute of Medical Sciences, Nagpur, IND; 2 General Medicine, All India Institute of Medical Sciences, Nagpur, IND; 3 Obstetrics and Gynaecology, Apex Fertility Centre, Nagpur, IND

**Keywords:** cardiovascular disease, stress, healthy heart diet, physical activity, lifestyle modifications, cardiovascular health

## Abstract

Cardiovascular diseases (CVDs) remain a leading cause of morbidity and mortality worldwide. Lifestyle modifications have gained increasing recognition as key interventions in preventing and managing CVDs. This narrative review aims to provide a thorough assessment of the impact of lifestyle modifications on cardiovascular health. The review encompasses various aspects, including diet, physical activity, smoking cessation, stress management, and weight management. Additionally, the review explores the underlying mechanisms by which lifestyle modifications influence cardiovascular health and highlights the evidence from clinical trials, observational studies, and meta-analyses. The findings of this review emphasize the importance of lifestyle modifications in reducing the risk factors associated with CVDs and improving cardiovascular outcomes.

## Introduction and background

Cardiovascular diseases (CVDs) pose a significant global burden in terms of morbidity, mortality, and economic impact. CVDs are highly prevalent worldwide [1]. According to the World Health Organization (WHO), CVDs are the leading cause of death globally, accounting for approximately 17.9 million deaths in 2019, representing 32% of all global deaths [2]. Moreover, CVDs affect individuals across all regions, countries, and socio-economic groups. They contribute to a substantial number of deaths each year [1]. Ischemic heart disease and stroke are the most common types of CVDs causing mortality. Low- and middle-income countries are particularly affected by the burden of CVD-related deaths [3]. Several risk factors contribute to the development of CVDs. These include hypertension, dyslipidemia, smoking, obesity, diabetes, physical inactivity, unhealthy diet, excessive alcohol consumption, and psychosocial factors such as stress [4]. The presence of multiple risk factors further amplifies the risk of developing CVDs. They also impose a substantial economic burden on healthcare systems and societies.

Lifestyle modifications serve as crucial preventive measures for CVDs. Risk factors associated with CVDs are modifiable and can be influenced through lifestyle modifications. Lifestyle modifications, such as adopting a healthy diet, engaging in regular physical activity, quitting smoking, managing stress, and maintaining a healthy weight, directly target these risk factors. By addressing modifiable risk factors, individuals can significantly reduce their risk of developing CVDs [5]. Lifestyle modifications offer a comprehensive approach to CVD prevention. Unlike pharmaceutical interventions that target specific pathways or risk factors, lifestyle modifications impact multiple risk factors simultaneously. Lifestyle modifications provide long-term benefits for cardiovascular health. By adopting healthy habits early in life and maintaining them over time, individuals can significantly reduce their risk of developing CVDs in the future. These modifications have a cumulative effect on cardiovascular health, resulting in sustained improvements and a lower risk of adverse events over time [6]. Lifestyle modifications complement medical interventions for CVDs. In many cases, lifestyle modifications can reduce the reliance on medications or enhance their effectiveness. For individuals already diagnosed with CVDs, lifestyle modifications serve as essential components of disease management, improving outcomes and quality of life.

## Review

Impact of diet on cardiovascular health

Role of a Heart-Healthy Diet in Reducing Cardiovascular Risk

A heart-healthy diet plays a critical role in reducing cardiovascular risk. A heart-healthy diet helps lower levels of low-density lipoprotein (LDL). High LDL is a major risk factor for the development of atherosclerosis. By reducing dietary intake of saturated and trans fats, a heart-healthy diet can help maintain optimal cholesterol levels and prevent the formation of plaque [7]. A heart-healthy diet promotes the control of blood pressure, another significant risk factor for CVDs. A diet rich in fruits, vegetables, whole grains, and low-fat dairy products, known as the Dietary Approaches to Stop Hypertension (DASH) diet, has been shown to effectively lower blood pressure. It emphasizes the consumption of foods that are low in sodium, high in potassium, magnesium, and calcium, which collectively contribute to maintaining healthy blood pressure levels [8]. Chronic inflammation plays a crucial role in the development and progression of CVDs. A heart-healthy diet, particularly one that includes anti-inflammatory foods, can help reduce inflammation in the body. Foods such as fatty fish (rich in omega-3 fatty acids), nuts, seeds, whole grains, fruits, and vegetables possess anti-inflammatory properties. By incorporating these foods into the diet, inflammation markers can be reduced, promoting cardiovascular health [9].

Maintaining a healthy weight is essential for reducing cardiovascular risk. A heart-healthy diet focuses on nutrient-dense foods that are lower in calories, unhealthy fats, and added sugars. It emphasizes portion control and encourages the consumption of lean proteins, fiber-rich foods, and healthy fats. Such dietary patterns, combined with regular physical activity, promote weight management and help prevent obesity, a significant risk factor for CVDs [10]. The long-term benefits include adopting a heart-healthy diet and making it lifelong have sustained benefits for cardiovascular health. Consistently following a heart-healthy diet can help prevent the development of CVDs, reduce the risk of heart attacks, strokes, and other cardiac events, and contribute to overall longevity and well-being.

Review of Evidence Supporting the Mediterranean Diet, DASH Diet, and Other Dietary Patterns

The Mediterranean diet and the DASH diet are two well-studied dietary patterns that have demonstrated significant benefits for cardiovascular health. Here is a summary of the evidence supporting these diets, as well as other dietary patterns.

Mediterranean diet: The Mediterranean diet refers to a dietary pattern inspired by the traditional eating habits of people living in Mediterranean regions, such as Greece, Italy, and Spain. It is characterized by an abundance of plant-based foods, including fruits, vegetables, whole grains, legumes, nuts, and seeds. Olive oil is the primary source of fat in this diet. Moderate consumption of fish and poultry is encouraged, with red meat being consumed in smaller quantities. The PREDIMED study (Prevención con Dieta Mediterránea) found that individuals who followed a Mediterranean diet supplemented with extra-virgin olive oil or mixed nuts had a 30% reduction in the risk of cardiovascular events compared to a control group following a low-fat diet [11]. Meta-analyses have shown that adherence to the Mediterranean diet is associated with a reduced risk of CVDs, including coronary heart disease, stroke, and overall mortality [12]. The Mediterranean diet has been found to improve lipid profiles by reducing LDL cholesterol levels and increasing high-density lipoprotein (HDL) cholesterol levels. It also has beneficial effects on blood pressure, endothelial function, inflammation markers, and insulin sensitivity [13].

DASH diet: The DASH diet, which emphasizes fruits, vegetables, whole grains, low-fat dairy products, lean proteins, and limited sodium intake, has been extensively studied for its impact on blood pressure. The original DASH trial demonstrated that individuals following the DASH diet experienced significant reductions in blood pressure, particularly among those with hypertension [14]. Subsequent studies have shown that the DASH diet can also improve lipid profiles, reduce the risk of CVDs, and lower the risk of developing type 2 diabetes [15].

Plant-based diets: Plant-based diets, such as vegetarian and vegan diets, have been associated with a lower risk of CVDs. A systematic review and meta-analysis showed that vegetarian and vegan diets are associated with lower blood pressure levels, improved lipid profiles, reduced body weight, and lower risk of ischemic heart disease compared to non-vegetarian diets [16]. The EPIC-Oxford study found that vegetarians had a 13% lower risk of developing ischemic heart disease compared to meat-eaters [17].

Dietary approaches: The American Heart Association (AHA) has recommended dietary approaches for cardiovascular health, including the combination of a heart-healthy diet (such as the Mediterranean or DASH diet) with reduced sodium intake, limited added sugars, and avoidance of trans fats. The AHA's "Life's Simple 7" guidelines, which include diet, physical activity, smoking cessation, weight management, blood pressure control, cholesterol management, and glucose control, have been associated with a significantly reduced risk of CVDs [18]. It is worth noting that while these dietary patterns have demonstrated benefits for cardiovascular health, individual variations, cultural differences, and specific health conditions should be taken into account when implementing dietary recommendations.

Effect of Specific Nutrients on Cardiovascular Outcomes

Specific nutrients play a significant role in cardiovascular outcomes. Omega-3 fatty acids, particularly eicosapentaenoic acid (EPA) and docosahexaenoic acid (DHA) found in fatty fish, have been associated with a reduced risk of cardiovascular events. They have shown benefits in reducing triglyceride levels, lowering blood pressure, improving endothelial function, and reducing inflammation [19]. The AHA recommends consuming fatty fish at least twice a week or considering omega-3 supplements for individuals with elevated triglyceride levels. Dietary fiber, especially soluble fiber, has been linked to improved cardiovascular health. High-fiber diets have shown benefits in reducing LDL cholesterol levels, improving glycemic control, and lowering the risk of developing CVDs [20]. Foods rich in fiber include whole grains, fruits, vegetables, legumes, and nuts.

Antioxidants, such as vitamins C and E, beta-carotene, and polyphenols, have been associated with cardiovascular benefits. They help reduce oxidative stress and inflammation, which are involved in the development of CVDs [21]. Foods rich in antioxidants include berries, citrus fruits, nuts, seeds, dark chocolate, and green leafy vegetables. Potassium and magnesium are minerals that play a role in maintaining healthy blood pressure levels. Adequate intake of these minerals has been associated with a lower risk of hypertension and improved cardiovascular health [22]. Potassium-rich foods include bananas, oranges, potatoes, tomatoes, and legumes. Magnesium-rich foods include leafy green vegetables, nuts, seeds, and whole grains. Excessive sodium intake has been associated with increased blood pressure and cardiovascular risk. Reducing sodium intake is recommended to maintain healthy blood pressure levels. Limiting processed and packaged foods, reading food labels, and cooking meals from scratch can help reduce sodium intake. Plant sterols and stanols are natural compounds found in fruits, vegetables, nuts, and seeds. They have been shown to lower LDL cholesterol levels by reducing its absorption in the intestine. Foods fortified with plant sterols or stanols, such as certain margarine, yogurts, and spreads, can be beneficial in reducing cholesterol levels [23]. Nutrients work synergistically within the context of a balanced diet. Rather than focusing on individual nutrients, a comprehensive approach that includes a variety of nutrient-rich foods is recommended for optimal cardiovascular health.

**Figure 1:**
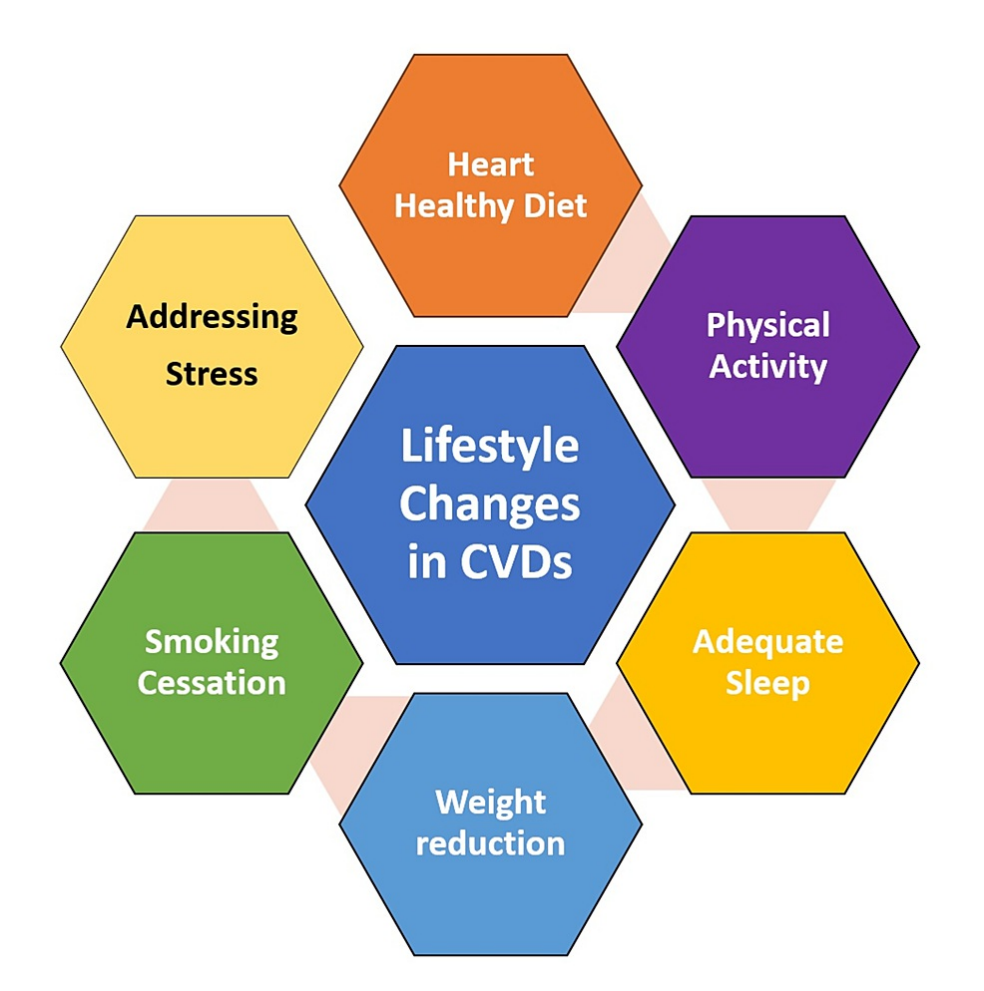
Lifestyle changes in cardiovascular disease CVDs: cardiovascular diseases. Image credits: Gunjan K. Ghodeshwar (Corresponding Author).

The lifestyle changes which shall impact cardiovascular health are depicted in Figure 1.

Physical activity and cardiovascular health

Relationship Between Physical Activity and Cardiovascular Risk Reduction

Physical activity plays a crucial role in reducing cardiovascular risk. Regular physical activity helps lower blood pressure, which is a major risk factor for CVDs. Engaging in aerobic exercises, such as brisk walking, jogging, cycling, or swimming, can lead to a decrease in both systolic and diastolic blood pressure. It improves the efficiency of the heart and blood vessels, reducing the strain on the cardiovascular system [24]. Physical activities have a positive impact on lipid profiles, including reducing levels of total cholesterol, LDL cholesterol, and triglycerides, while increasing levels of HDL cholesterol. This favorable lipid profile can help prevent the development of atherosclerosis and reduces the risk of CVDs [25].

Regular physical activity contributes to weight management and helps maintain a healthy body weight. Obesity is a significant risk factor for CVDs, and losing excess weight or preventing weight gain through physical activity reduces the risk of developing cardiovascular conditions such as heart disease, stroke, and type 2 diabetes [26]. Physical activity improves insulin sensitivity, which is important for maintaining stable blood sugar levels. Regular exercise helps the body use insulin effectively, reducing the risk of insulin resistance, metabolic syndrome, and type 2 diabetes, which are closely linked to an increased risk of CVDs [27].

Chronic inflammation is involved in the development and progression of CVDs. Regular physical activity has anti-inflammatory effects, reducing levels of inflammatory markers in the body. By reducing inflammation, physical activity helps protect the blood vessels and lowers the risk of cardiovascular events [28]. Physical activity improves endothelial function. It promotes the release of nitric oxide, a substance that helps dilate blood vessels, improving blood flow and reducing the risk of blood clots and plaque formation [29]. Physical activity acts as a natural stress reliever. Regular exercise reduces stress levels, promotes mental well-being, and helps manage psychosocial factors that contribute to cardiovascular risk. High levels of stress can negatively impact cardiovascular health, and physical activity provides a healthy outlet for stress management [30].

It is important to note that the beneficial effects of physical activity on cardiovascular health are dose-dependent, meaning that higher levels of activity often lead to greater risk reduction. The AHA recommends at least 150 minutes of moderate-intensity aerobic exercise or 75 minutes of vigorous-intensity aerobic exercise per week, along with muscle-strengthening activities at least two days a week, for optimal cardiovascular health [31].

Effects of Aerobic Exercise, Resistance Training, and Combined Training on Cardiovascular Outcomes

Aerobic exercise, resistance training, and combined training (a combination of aerobic and resistance exercises) each have distinct effects on cardiovascular outcomes. Aerobic exercise, also known as cardiovascular or endurance exercise, includes activities that increase the heart rate and promote cardiovascular fitness. Regular aerobic exercise improves cardiovascular health by strengthening the heart muscle, increasing the heart's efficiency, and enhancing blood flow throughout the body. Benefits of aerobic exercise include a reduction in resting heart rate, improved oxygen uptake, increased cardiac output, and improved blood vessel function. Aerobic exercise has been associated with a lower risk of developing CVDs, such as coronary heart disease, stroke, and heart failure. It increases the levels of HDL cholesterol and reduces total cholesterol, LDL cholesterol, and triglycerides. Aerobic exercise also helps control blood pressure, reduces inflammation, improves insulin sensitivity, and promotes weight management [24].

Resistance training, also known as strength or weight training, involves using resistance or weights to strengthen muscles. While resistance training primarily focuses on muscular strength and endurance, it also has cardiovascular benefits. Resistance training can improve cardiovascular health by reducing resting blood pressure, increasing HDL cholesterol levels, and improving insulin sensitivity. It enhances overall body composition by increasing muscle mass and reducing body fat, which contributes to improved cardiovascular risk factors. Resistance training can have a positive impact on arterial stiffness, a measure of blood vessel health, and improve endothelial function. Combining resistance training with aerobic exercise has been found to have additive effects on cardiovascular outcomes, providing a comprehensive approach to cardiovascular health [32].

Combined training refers to the integration of both aerobic exercise and resistance training into a single exercise program. This approach provides the benefits of both aerobic and resistance exercises, leading to improved cardiovascular health and overall fitness. Combined training has been shown to be effective in reducing blood pressure, improving lipid profiles, and enhancing insulin sensitivity. It can lead to greater improvements in body composition, including increased muscle mass and decreased body fat, compared to either aerobic or resistance training alone. Combined training is particularly beneficial for individuals aiming to achieve overall cardiovascular fitness, maintain a healthy weight, and manage multiple cardiovascular risk factors [33].

Recommended Exercise Guidelines for Cardiovascular Health

The AHA provides exercise guidelines for cardiovascular health [31].

Aerobic exercise: Adults should aim for at least 150 minutes of moderate-intensity aerobic exercise or 75 minutes of vigorous-intensity aerobic exercise per week. Moderate-intensity aerobic activities include brisk walking, cycling at a moderate pace, swimming, or dancing. Vigorous-intensity activities include running, cycling at a high speed, aerobic dancing, or playing sports like soccer or basketball. These aerobic exercises can be performed in bouts of 10 minutes or more throughout the week. For additional health benefits, adults can increase the aerobic exercise duration to 300 minutes of moderate-intensity or 150 minutes of vigorous-intensity per week [31].

Resistance training: Adults should include strength training exercises at least two days per week. Focus on exercises that target major muscle groups, including the legs, hips, back, abdomen, chest, shoulders, and arms. Perform a variety of exercises using body weight, resistance bands, free weights, or weight machines. Aim for eight to 12 repetitions of each exercise, gradually increasing the resistance over time [31].

Flexibility and balance exercises: Include flexibility exercises at least two to three days per week, focusing on major muscle-tendon groups. Stretching exercises, yoga, or Pilates can help improve flexibility. For older adults at risk of falls, balance exercises should be incorporated into the routine. Examples include standing on one foot, walking heel-to-toe, or tai chi [31].

Sedentary behavior: Reduce sedentary behavior and aim to minimize the amount of time spent sitting. Incorporate breaks from sitting by standing or walking for a few minutes every hour [31].

Smoking cessation and cardiovascular health

Smoking is a well-established risk factor for CVDs, and the harmful effects of smoking on the cardiovascular system are mediated through various mechanisms such as endothelial dysfunction, inflammation, oxidative stress, lipid abnormalities, platelet activation, thrombosis, increased blood pressure, coronary spasm, and impaired cardiac function. It is worth noting that the harmful effects of smoking on the cardiovascular system are not limited to active smokers. Second-hand smoke exposure (inhalation of smoke from others) also poses risks to cardiovascular health [34]. Quitting smoking significantly reduces the risk of developing CVDs and improves overall health by reducing the risk of atherosclerosis, improving blood pressure, positive change in lipid profile, decreasing blood clotting tendency, and reducing inflammation and oxidative stress. The benefits of smoking cessation are substantial, and individuals who quit smoking experience a gradual reduction in their cardiovascular risk over time. It is important to note that the benefits of smoking cessation on cardiovascular health are significant regardless of the duration or intensity of prior smoking. Even individuals who have smoked for many years or who have developed smoking-related health conditions can experience substantial improvements in their cardiovascular risk profile by quitting smoking [35].

Approaches and Interventions for Smoking Cessation

There are several approaches and interventions available to support individuals in their journey toward smoking cessation, such as behavioral counseling, medications, combination therapy, motivational interviews, mobile apps and online programs, support groups, mindfulness and stress reduction techniques, and tailored interventions based on specific circumstances. Quitting smoking is a highly individual process, and different strategies may work better for different people. Combining multiple approaches, staying motivated, and persisting in the face of challenges can greatly increase the chances of successful smoking cessation [36].

Stress management and cardiovascular health

Chronic stress plays a significant role in the development and progression of CVDs by activation of the sympathetic nervous system, leading to a state of chronic inflammation in the body, dysregulation of the hypothalamic-pituitary-adrenal (HPA) axis, changes in blood clotting and platelet function, and unhealthy coping behaviors. It impacts sleep and contributes to psychological factors such as depression, anxiety, and social isolation. It is important to note that chronic stress does not act in isolation, and its impact on cardiovascular health can interact with other risk factors, such as smoking, obesity, and genetics, amplifying the overall risk. Effective stress management techniques, such as regular exercise, relaxation techniques (e.g., meditation and deep breathing exercises), social support, and seeking professional help when needed, can help mitigate the detrimental effects of chronic stress on cardiovascular health [37].

There are several techniques for stress reduction that have been shown to have positive effects on cardiovascular outcomes, such as relaxation techniques, regular physical exercise, building and maintaining strong social connections and support networks, cognitive-behavioral therapy (CBT), mindfulness-based stress reduction (MBSR), biofeedback techniques that involve using electronic devices to monitor physiological signals, such as heart rate, blood pressure, or muscle tension, and providing real-time feedback. It is important to note that the effectiveness of these techniques may vary among individuals, and it may be beneficial to explore a combination of approaches to find what works best for each person [38].

Clinical evidence and recommendations

There is a wealth of evidence from clinical trials, observational studies, and meta-analyses supporting the positive impact of lifestyle modifications on various aspects of health, including cardiovascular health. Diabetes Prevention Program (DPP), a landmark clinical trial, demonstrated that lifestyle interventions, including diet modification and increased physical activity, were effective in reducing the incidence of type 2 diabetes in high-risk individuals [39]. DASH-Sodium Trial showed that the DASH diet, which emphasizes fruits, vegetables, whole grains, and low-fat dairy products, combined with sodium reduction, significantly lowered blood pressure [40]. Look AHEAD Trial investigated the effects of lifestyle interventions, including weight loss, healthy eating, and increased physical activity, on cardiovascular outcomes in overweight or obese individuals with type 2 diabetes. It showed that intensive lifestyle interventions resulted in modest weight loss and improved cardiovascular risk factors [41]. Long-term observational studies such as the Nurses' Health Study and Health Professionals Follow-up Study have provided valuable insights into the relationship between lifestyle factors (such as diet, physical activity, smoking, and alcohol consumption) and the risk of CVDs, diabetes, and other chronic conditions [42].

Current guidelines and recommendations for lifestyle modifications in cardiovascular health

The American Heart Association (AHA) Guidelines

Diet: The AHA recommends a heart-healthy diet that emphasizes fruits, vegetables, whole grains, lean proteins, and low-fat dairy products. It encourages limiting saturated and trans fats, sodium, and added sugars.

Physical activity: The AHA recommends at least 150 minutes of moderate-intensity aerobic activity or 75 minutes of vigorous-intensity aerobic activity per week, along with muscle-strengthening activities at least two days per week.

Smoking cessation: The AHA strongly advises against smoking and supports comprehensive smoking cessation programs [31].

The European Society of Cardiology (ESC) Guidelines

Lifestyle modifications: The ESC guidelines emphasize lifestyle modifications as the foundation for CVD prevention. These include a healthy diet, regular physical activity, smoking cessation, and weight management.

Dietary patterns: The ESC recommends the Mediterranean diet as an optimal dietary pattern for cardiovascular health due to its emphasis on fruits, vegetables, whole grains, legumes, fish, and olive oil.

Physical activity: The ESC recommends at least 150 minutes of moderate-intensity aerobic exercise or 75 minutes of vigorous-intensity aerobic exercise per week, along with muscle-strengthening activities [43].

The World Health Organization (WHO) Recommendations

Diet: The WHO recommends a balanced diet that includes a variety of fruits, vegetables, whole grains, lean proteins, and healthy fats. It advises reducing the intake of free sugars, salt, and saturated and trans fats.

Physical activity: The WHO recommends at least 150 minutes of moderate-intensity aerobic activity or 75 minutes of vigorous-intensity aerobic activity per week, along with muscle-strengthening activities.

Tobacco control: The WHO advocates for comprehensive tobacco control policies, including tobacco taxes, smoke-free environments, and cessation support [44].

## Conclusions

Lifestyle modifications have a profound impact on cardiovascular health. Implementing healthy habits, such as regular physical activity, a balanced diet, smoking cessation, stress management, and adequate sleep, can significantly reduce the risk of CVDs and improve overall cardiovascular well-being. This review underscores the need for a comprehensive approach to lifestyle modifications. Combining multiple healthy habits yields synergistic effects on cardiovascular health. Adoption of these lifestyle modifications should be sustained over the long term to maximize their benefits. While the evidence presented in this review is compelling, it is essential to acknowledge that individual factors, such as genetics and underlying medical conditions, can influence the effectiveness of lifestyle modifications on cardiovascular health. Therefore, personalized approaches, tailored to each individual's unique circumstances, are warranted.

## References

[1] Vaduganathan M, Mensah GA, Turco JV, Fuster V, Roth GA (2022). The global burden of cardiovascular diseases and risk: a compass for future health. J Am Coll Cardiol.

[2] (2021). World Health Organization. Cardiovascular diseases (CVDs). https://www.who.int/news-room/fact-sheets/detail/cardiovascular-diseases-(cvds)#:~:text=Cardiovascular%20diseases%20(CVDs)%20are%20the,%2D%20and%20middle%2Dincome%20countries..

[3] Wurie HR, Cappuccio FP (2012). Cardiovascular disease in low- and middle-income countries: an urgent priority. Ethn Health.

[4] (2023). CDC. Know your risk for heart disease. https://www.cdc.gov/heartdisease/risk_factors.htm.

[5] Rippe JM (2019). Lifestyle strategies for risk factor reduction, prevention, and treatment of cardiovascular disease. Am J Lifestyle Med.

[6] Aggarwal M, Bozkurt B, Panjrath G (2018). Lifestyle modifications for preventing and treating heart failure. J Am Coll Cardiol.

[7] Feingold KR (2021). The effect of diet on cardiovascular disease and lipid and lipoprotein levels. Endotext.

[8] Challa HJ, Ameer MA, Uppaluri KR (2023). DASH Diet To Stop Hypertension. https://www.ncbi.nlm.nih.gov/books/NBK482514/.

[9] Stromsnes K, Correas AG, Lehmann J, Gambini J, Olaso-Gonzalez G (2021). Anti-inflammatory properties of diet: role in healthy aging. Biomedicines.

[10] De Bacquer D, Jennings CS, Mirrakhimov E (2022). Potential for optimizing management of obesity in the secondary prevention of coronary heart disease. Eur Heart J Qual Care Clin Outcomes.

[11] Estruch R, Ros E, Salas-Salvadó J (2018). Primary prevention of cardiovascular disease with a Mediterranean diet supplemented with extra-virgin olive oil or nuts. N Engl J Med.

[12] Sofi F, Macchi C, Abbate R, Gensini GF, Casini A (2014). Mediterranean diet and health status: an updated meta-analysis and a proposal for a literature-based adherence score. Public Health Nutr.

[13] Schwingshackl L, Morze J, Hoffmann G (2020). Mediterranean diet and health status: active ingredients and pharmacological mechanisms. Br J Pharmacol.

[14] Tyson CC, Nwankwo C, Lin PH, Svetkey LP (2012). The Dietary Approaches to Stop Hypertension (DASH) eating pattern in special populations. Curr Hypertens Rep.

[15] Campbell AP (2017). DASH eating plan: an eating pattern for diabetes management. Diabetes Spectr.

[16] Dybvik JS, Svendsen M, Aune D (2023). Vegetarian and vegan diets and the risk of cardiovascular disease, ischemic heart disease and stroke: a systematic review and meta-analysis of prospective cohort studies. Eur J Nutr.

[17] Tong TY, Appleby PN, Bradbury KE, Perez-Cornago A, Travis RC, Clarke R, Key TJ (2019). Risks of ischaemic heart disease and stroke in meat eaters, fish eaters, and vegetarians over 18 years of follow-up: results from the prospective EPIC-Oxford study. BMJ.

[18] Lichtenstein AH, Appel LJ, Vadiveloo M (2021). 2021 dietary guidance to improve cardiovascular health: a scientific statement from the American Heart Association. Circulation.

[19] Swanson D, Block R, Mousa SA (2012). Omega-3 fatty acids EPA and DHA: health benefits throughout life. Adv Nutr.

[20] McRae MP (2017). Dietary fiber is beneficial for the prevention of cardiovascular disease: an umbrella review of meta-analyses. J Chiropr Med.

[21] Leopold JA (2015). Antioxidants and coronary artery disease: from pathophysiology to preventive therapy. Coron Artery Dis.

[22] Karppanen H (1991). Minerals and blood pressure. Ann Med.

[23] Salehi B, Quispe C, Sharifi-Rad J (2020). Phytosterols: from preclinical evidence to potential clinical applications. Front Pharmacol.

[24] Nystoriak MA, Bhatnagar A (2018). Cardiovascular effects and benefits of exercise. Front Cardiovasc Med.

[25] Wang Y, Xu D (2017). Effects of aerobic exercise on lipids and lipoproteins. Lipids Health Dis.

[26] Cercato C, Fonseca FA (2019). Cardiovascular risk and obesity. Diabetol Metab Syndr.

[27] Bird SR, Hawley JA (2016). Update on the effects of physical activity on insulin sensitivity in humans. BMJ Open Sport Exerc Med.

[28] Cerqueira É, Marinho DA, Neiva HP, Lourenço O (2019). Inflammatory effects of high and moderate intensity exercise—a systematic review. Front Physiol.

[29] Gao J, Pan X, Li G, Chatterjee E, Xiao J (2022). Physical exercise protects against endothelial dysfunction in cardiovascular and metabolic diseases. J Cardiovasc Transl Res.

[30] Franklin BA, Rusia A, Haskin-Popp C, Tawney A (2021). Chronic stress, exercise and cardiovascular disease: placing the benefits and risks of physical activity into perspective. Int J Environ Res Public Health.

[31] Piercy KL, Troiano RP, Ballard RM (2018). The physical activity guidelines for Americans. JAMA.

[32] Braith RW, Stewart KJ (2006). Resistance exercise training: its role in the prevention of cardiovascular disease. Circulation.

[33] Liang M, Pan Y, Zhong T, Zeng Y, Cheng AS (2021). Effects of aerobic, resistance, and combined exercise on metabolic syndrome parameters and cardiovascular risk factors: a systematic review and network meta-analysis. Rev Cardiovasc Med.

[34] Roy A, Rawal I, Jabbour S, Prabhakaran D (2017). Tobacco and cardiovascular disease: a summary of evidence. Cardiovascular, Respiratory, and Related Disorders.

[35] Gallucci G, Tartarone A, Lerose R, Lalinga AV, Capobianco AM (2020). Cardiovascular risk of smoking and benefits of smoking cessation. J Thorac Dis.

[36] Engstrom PF, Clapper ML, Schnoll RA (2003). Approaches for smoking cessation. Holland-Frei Cancer Medicine.

[37] Chinnaiyan KM (2019). Role of stress management for cardiovascular disease prevention. Curr Opin Cardiol.

[38] Rainforth MV, Schneider RH, Nidich SI, Gaylord-King C, Salerno JW, Anderson JW (2007). Stress reduction programs in patients with elevated blood pressure: a systematic review and meta-analysis. Curr Hypertens Rep.

[39] Diabetes Prevention Program (DPP) Research Group (2002). The Diabetes Prevention Program (DPP): description of lifestyle intervention. Diabetes Care.

[40] Svetkey LP, Sacks FM, Obarzanek E (1999). The DASH Diet, Sodium Intake and Blood Pressure Trial (DASH-Sodium): rationale and design. J Am Diet Assoc.

[41] Pi-Sunyer X (2014). The Look AHEAD Trial: a review and discussion of its outcomes. Curr Nutr Rep.

[42] Hu FB, Willett WC (2001). Diet and coronary heart disease: findings from the Nurses' Health Study and Health Professionals' Follow-up Study. J Nutr Health Aging.

[43] Visseren FL, Mach F, Smulders YM (2021). 2021 ESC guidelines on cardiovascular disease prevention in clinical practice. Eur Heart J.

[44] World Health Organization (2007). World Health Organization. Prevention of cardiovascular disease: guidelines for assessment and management of total cardiovascular risk. https://www.who.int/publications/i/item/prevention-of-cardiovascular-disease---guidelines-for-assessment-and-management-of-total-cardiovascular-risk.

